# Full retraction: Azza E. B. El-Remessy, Ahmad M. Rabie, Mamdouh M. El-Shishtawy, Laila A. Eissa, Gregory I. Liou. Regulation of interphotoreceptor retinoid-binding protein (IRBP) gene expression by cAMP in differentiated retinoblastoma cells. Mol Vis. 2000; 6:243-251.

**Published:** 2014-09-08

The authors made substantive errors in figure images of this article such that the hypotheses were not tested and the conclusions were not supported.

On this basis, the Editors formally retract this article from Molecular Vision.

Sincerely,

The Editors of Molecular Vision

